# Peripherally inserted central catheter versus central venous catheter for intravenous access: A protocol for systematic review and meta-analysis

**DOI:** 10.1097/MD.0000000000020352

**Published:** 2020-07-24

**Authors:** Felipe Kenzo Yadoya Santos, Ronald Luiz Gomes Flumignan, Libnah Leal Areias, Anna Karina Paiva Sarpe, Fabio Cabral Freitas Amaral, Rafael Bernardes de Ávila, Vladimir Tonello de Vasconcelos, Henrique Jorge Guedes Neto, Jorge Eduardo de Amorim, Luis Carlos Uta Nakano

**Affiliations:** a Undergraduate student of medicine; b Division of Vascular and Endovascular Surgery, Department of Surgery, Escola Paulista de Medicina, Universidade Federal de São Paulo, São Paulo-SP, Brazil.

**Keywords:** central venous catheters, clinical protocols, evidence-based medicine, peripheral catheterization, systematic review

## Abstract

**Background:**

Since the first description of the central venous catheter (CVC) in 1952, it has been used for the rapid administration of drugs, chemotherapy, as a route for nutritional support, blood components, monitoring patients, or combinations of these. When CVC is used in the traditional routes (eg, subclavian, jugular, and femoral veins), the complication rates range up to 15% and are mainly due to mechanical dysfunction, infection, and thrombosis. The peripherally inserted central catheter (PICC) is an alternative option for CVC access. However, the clinical evidence for PICC compared to CVC is still under discussion. In this setting, this systematic review (SR) aims to assess the effects of PICC compared to CVC for intravenous access.

**Methods:**

We will perform a comprehensive search for randomised controlled trials (RCTs), which compare PICC and traditional CVC for intravenous access. The search strategy will consider free text terms and controlled vocabulary (eg, MeSH and Entree) related to “peripherally inserted central venous catheter,” “central venous access,” “central venous catheter,” “catheterisation, peripheral,” “vascular access devices,” “infusions, intravenous,” “administration, intravenous,” and “injections, intravenous.” Searches will be carried out in these databases: MEDLINE (via PubMed), EMBASE (via Elsevier), Cochrane CENTRAL (via Wiley), IBECS, and LILACS (both via Virtual Health Library). We will consider catheter-related deep venous thrombosis and overall successful insertion rates as primary outcomes and haematoma, venous thromboembolism, reintervention derived from catheter dysfunction, catheter-related infections, and quality of life as secondary outcomes. Where results are not appropriate for a meta-analysis using RevMan 5 software (eg, if the data have considerable heterogeneity and are drawn from different comparisons), a descriptive analysis will be performed.

**Results:**

Our SR will be conducted according to the Cochrane Handbook of Systematic Reviews of Interventions and the findings will be reported in compliance with PRISMA.

**Conclusion:**

Our study will provide evidence for the effects of PICC versus CVC for venous access.

**Ethics and dissemination:**

This SR has obtained formal ethical approval and was prospectively registered in Open Science Framework. The findings of this SR will be disseminated through peer-reviewed publications or conference presentations.

**Registration:**

osf.io/xvhzf.

**Ethical approval:**

69003717.2.0000.5505.

## 1 Introduction

Millions of catheters are implanted annually in health services to provide support for management of acute and chronic diseases in adults and pediatric patients.^[1]^ Central venous access can be performed by a central venous catheter (CVC) or peripherally inserted central catheter (PICC) and the indications include intravenous therapy, access to the central venous system for multiple procedures, blood sample collection, and monitoring, mainly when peripheral venous access is inaccessible.^[1–3]^ There are many types of devices available and the choice mainly depends on the estimated time required for use (short, medium, or long term).^[1]^ PICCs are usually recommended for short and medium-term use (4 weeks to 6 months).^[1]^

PICC use has increased in recent years, especially in oncologic and critical patients as an alternative to CVC, mainly due to the easier insertion, safety, and possible cost-effectiveness advantages.^[4–6]^ Other possible benefits of PICC are the virtual elimination of the high morbidity and potentially fatal complications of neck and chest catheter insertions, the reduced potential for catheter-related infection and septicemia, and lower procedure costa.^[7,8]^ In addition, as it can be used for medium-term access, it is suitable for intravenous therapy in home care patients. However, complications include catheter-associated thrombosis (3%), mechanical complications (4%), catheter-associated bloodstream infections (2%), and cellulitis (1%).^[9]^

There is no clear superiority of any procedure type, PICC or CVC, for venous access.^[10,11]^ For instance, the Clinical Practice Guidelines of the European Society of Vascular Surgery reports that PICC is related to a higher risk of venous thrombosis and a lower risk of catheter occlusion than CVC.^[12]^ Nevertheless, this information is based on moderate certainty evidence with some risk of bias, according to GRADE^[13]^ (eg, prospective cohort study).^[14]^ Robust evidence comparing PICC and CVC in critically ill patients is urgently needed.

Intending to fill this lack of evidence, we aim to conduct a systematic review (SR) under the well-established Cochrane recommendations to assess the effects of PICC versus CVC for intravenous access.

## 2 Methods

The “Cochrane Handbook for systematic review of interventions” recommendations will guide this SR, which is prospectively registered in the Open Science Framework at osf.io/xvhzf.^[15]^ This SR has obtained formal ethical approval of the research ethics committee of the Universidade Federal de São Paulo (69003717.2.0000.5505). The Preferred Reporting Items for Systematic Review and Meta-Analysis Protocol (PRISMA-P) guided this SR protocol report.^[16]^

### 2.1 Types of studies

We will include all randomized controlled trials (RCTs) with a parallel (eg, cluster or individual) or cross-over design, reported as full text, published as abstract only and unpublished data. Quasi-RCTs, that is, the studies in which participants are allocated to intervention groups based on methods that are not truly random, such as date of birth or hospital number, will not be considered.

### 2.2 Types of participants

We will include people of any age and sex who required any form of central venous access (PICC or CVC) for diagnostic or therapeutic purposes. We will consider all aims for related venous access such as venous catheterization for cardiac interventions, pulmonary artery monitoring blood pressure, nutrition and medicine infusion. We will also consider all variations of PICC or CVC, such as different device caliper, number of lumens, site of insertion (eg, cephalic or basilic veins for PICC, femoral, jugular, or subclavian veins for CVC). If only a subset of the participants meet our inclusion criteria (eg, studies with mixed populations), we will attempt to obtain data for the subgroup of interest from the trialists to include the study.

### 2.3 Types of interventions

We will include trials comparing PICC versus CVC for intravenous access. We will consider all types of Seldinger techniques for venous access, such as anterior wall puncture, vein transfixation, “catheter over the needle,” and other special devices as the baseline eligible technique for venous catheterization, and we will evaluate their differences under the subgroup analysis and investigation of heterogeneity section.

The main comparison will be peripherally inserted CVC versus CVC.

### 2.4 Types of outcome measures

Reporting ≥1 of the outcomes listed here in the trials is not an inclusion criterion for the SR. Where a published report does not appear to report one of these outcomes, we will access the trial protocol and contact the trial authors to assess whether the outcomes are available but not reported. Relevant trials which measured these outcomes but did not report the data at all, or not in a usable format, will be included in the review as part of the narrative. Economic costs will be evaluated indirectly by outcomes such as “overall successful intervention rate” and “catheter-related infection.” Since this is not a cost-effectiveness review, we will treat direct costs’ data narratively in the discussion section, if these data are available.

We will present the outcomes at 2 different time points following the start of the intervention, if data are available. Our time point of primary interest is early, but we also plan to report the long-term outcomes at the longest possible time of follow-up:

1. Early outcomes (at ≤30 days after intervention)
2. Long-term outcomes (>30 days after intervention)

#### 2.4.1 Primary outcomes

1. Catheter-related deep venous thrombosis, confirmed by at least 1 additional objective method (eg, duplex ultrasound or angiography by tomography, magnetic resonance imaging or digital subtraction fluoroscopy).
2. Overall successful insertion rates, that is the number of participants in whom the proposed method of catheterization was successful.

#### 2.4.2 Secondary outcomes

1. Major haematoma, defined as those either requiring an intervention (eg, open surgical or percutaneous drainage) or prolonging duration of hospital stay. We will consider the total number of perioperative and postoperative major hematomas.
2. Venous thromboembolism (eg, deep vein thrombosis, pulmonary embolism): confirmed through complementary tests (eg, duplex ultrasound, computed tomography, magnetic resonance imaging, angiography, ventilation-perfusion lung scan).
3. Reintervention derived from catheter dysfunction, that is, any catheter dysfunction such as occlusion, obstruction, infection, perforation, or fixation loss.
4. Catheter-related infection.
5. Quality of life (QoL): participant's subjective perception of improvement (yes or no) as reported by the study authors, or using any validated scoring system such as the Short Form-36 Health Survey (SF-36).^[17]^

## 3 Search methods

### 3.1 Electronic searches

Searches will be carried out in the databases Medical Literature Analysis and Retrieval System Online (MEDLINE [via PubMed]), Excerpta Medica database (EMBASE [via Elsevier]), Cochrane Central Register of Controlled Trials (CENTRAL [via Wiley]), Indice Bibliográfico Español de Ciencias de la Salud (IBECS), and Latin American and Caribbean Centre on Health Sciences Information (LILACS) (both via Virtual Health Library). We will also search through the World Health Organization International Clinical Trials Registry Platform (apps.who.int/trialsearch/), the clinical trial registries of ClinicalTrials.gov for ongoing or unpublished trials. Electronic search strategies will be as sensitive as possible, with no limits of language, date, or publication status. A sample search strategy for MEDLINE via PubMed is presented in Table 1.

**Table 1:**
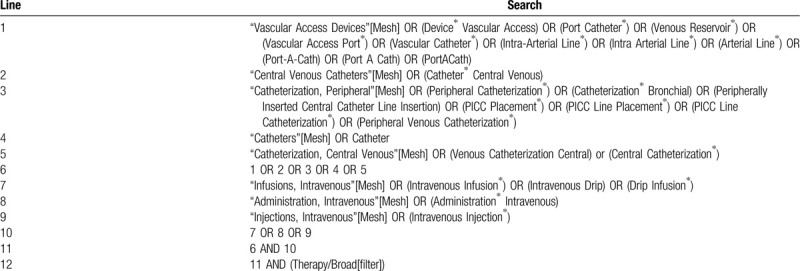
MEDLINE search strategy via PubMed.

### 3.2 Hand search and other search resources

We will also search for RCTs in the reference lists of all included studies to avoid missing important studies that may not have been indexed in electronic databases.

### 3.3 Selection of studies

Using the electronic tool covidence.org, 2 review authors (FKYS and RLGF) will independently evaluate the studies identified by the literature search and code them as eligible or not. We will resolve disagreements by discussion with the author team.

We will read the full text of the relevant trials and identify studies for inclusion. Moreover, we will identify and exclude duplicates and collate multiple reports of the same study so that each study, rather than each report, is the unit of interest in the review. If a trial does not meet the eligibility criteria, we will document the reasons for exclusion. The study selection will be illustrated in a PRISMA diagram.

### 3.4 Data extraction and management

Two review authors (FKYS and RLGF) will independently evaluate the trials to determine whether they are eligible for inclusion. We will resolve disagreements by discussion with the author team. The data extraction will include:

1. General features of the studies: author, year of publication, country, number of participants, age, sex of participants, and associated clinical conditions.
2. Specific features of the studies: intervention, comparison, primary and secondary outcomes, blinding, inclusion and exclusion criteria.

One reviewer (FKYS) will copy the data from the data collection form into the Review Manager (RevMan5.3) file for statistical analysis.^[18]^ A second review author (RLGF) will spot-check the study characteristics for accuracy against the trial report.

### 3.5 Assessment of risk of bias in included studies

Two review authors (FKYS and RLGF) will independently assess the included studies’ risk of bias using Cochrane's “Risk of bias” tool, described in Chapter 8 of the Cochrane Handbook for Systematic Reviews of interventions.^[19]^ The following aspects will be considered: random sequence generation, allocation concealment, blinding of participants and personnel, blinding of outcomes’ assessment, incomplete outcome data, selective reporting. and other sources of bias. Each component of the risk of bias tool in the included studies will be judged as a low, high, or unclear risk of bias. We will resolve disagreements by discussion within the review team.

### 3.6 Measures of treatment effect

We will use risk ratio (RR) for dichotomous data, mean difference for continuous data where the same scales or scores are used, and standardized mean difference for continuous data where different scales or scores are used, and all with 95% confidence intervals (CI).

We will calculate the number needed to treat (NNT) for the outcomes with direct implications for practice, using NNT = 1 / |risk difference|. The risk difference will be obtained with Review Manager 5 software. We will express the NNT as “number needed to treat for an additional beneficial outcome” and “number needed to treat for an additional harmful outcome” to indicate direction of effect.^[20]^

### 3.7 Unit of analysis issues

We will use an intention-to-treat approach and we will consider the participant as the unit of analysis for all outcomes.

### 3.8 Dealing with missing data

We will analyze the available data and intend to contact the trial authors to request missing data (eg, when a study is identified as an abstract only). Where possible, we will use the RevMan calculator to calculate missing standard deviations using other data from the trial (eg, CI). Where this is not possible, and the missing data are thought to introduce serious bias, we will explore the impact of including such studies in the overall assessment of results by a sensitivity analysis.

### 3.9 Assessment of heterogeneity

We will inspect forest plots visually to consider the direction and magnitude of effects and the degree of overlap between CIs. We will use the *I*^2^ statistic to measure heterogeneity among the trials in each analysis but acknowledge that there is substantial uncertainty in the value of *I*^2^ when there is only a small number of studies. We will also consider the *P* value from the *χ*^2^ test. If we identify substantial heterogeneity, we will report it and explore possible causes by prespecified subgroup analysis. We will use these ranges to guide our interpretation of the *I*^2^ statistic according to section 10.10.2 of the Cochrane Handbook for Systematic Reviews of Interventions.^[21]^

- 0% to 40%: heterogeneity might not be important,
- 30% to 60%: may represent moderate heterogeneity,
- 50% to 90%: may represent substantial heterogeneity,
- 75% to 100%: indicates considerable heterogeneity.

### 3.10 Assessment of reporting biases

We will assess the presence of publication bias and other reporting bias using funnel plots if sufficient studies (>10) are included in the meta-analysis.^[19]^

### 3.11 Data Synthesis

We will synthesize the data using RevMan 5.3 software.^[18]^ We will use the fixed-effect model to synthesize the data if there are low to moderate levels of heterogeneity. If there is substantial heterogeneity, we will use a random-effect model. If it is not appropriate to combine in a meta-analysis, we will not undertake a meta-analysis but will describe the data narratively. We plan to extract study data, format our comparisons in data tables, and prepare a summary of findings table before writing the results and conclusions of the review. A template summary of findings table is included as Table 2 .

**Table 2:**
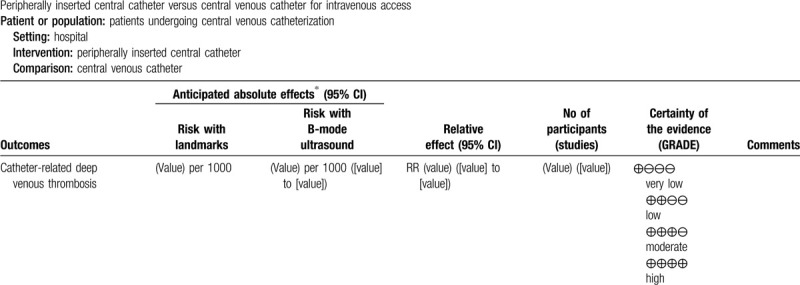
Summary of findings table.

**Table 2 (Continued):**
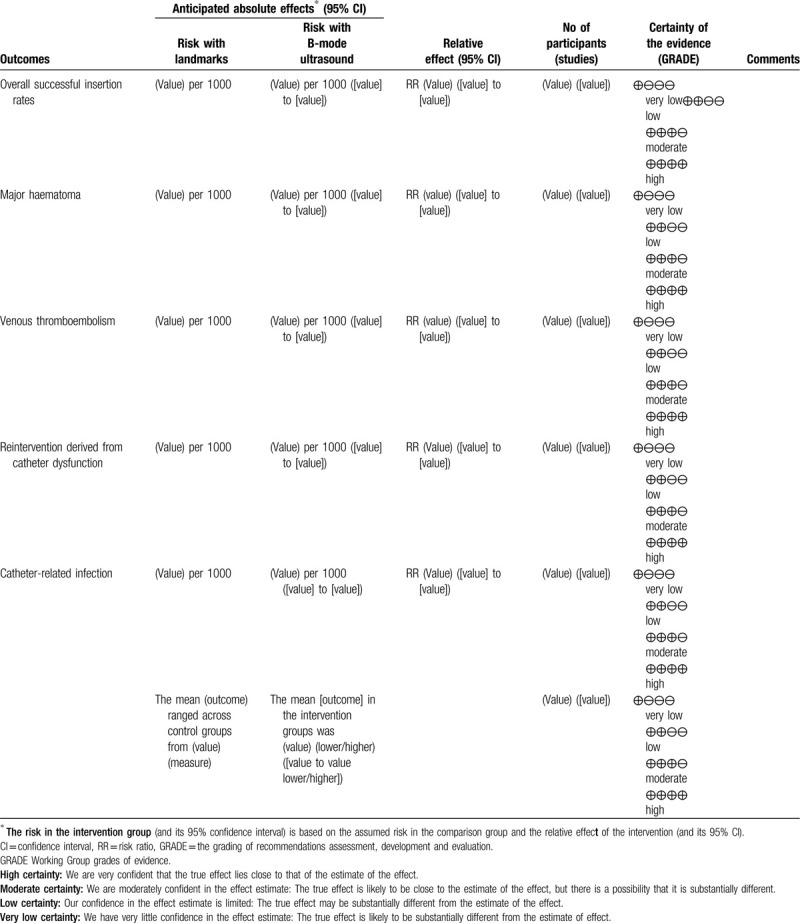
Summary of findings table.

### 3.12 “Summary of findings” table

A “summary of findings” will be made for each of the following outcomes: catheter-related deep venous thrombosis, overall successful insertion rates, major hematoma, venous thromboembolism, reintervention derived from catheter dysfunction, catheter-related infection, and QoL.

We will use the 5 GRADE domains (study limitations, consistency of effect, imprecision, indirectness, and publication bias) to assess the certainty of evidence relating to the studies which contribute data to the meta-analyses for the prespecified outcomes.^[22]^

### 3.13 Subgroup analysis and investigation of heterogeneity

Where there are sufficient data available, we will perform subgroup analyses for the following:

1. Participant characteristics: age (eg, children [until 17 years’ old], youth [18–24 years], adults [25–64 years], and seniors [≥65 years]), body mass index according to Table 3,^[23]^ comorbidities (eg, critically ill subjects, requiring vasopressors, electives procedures), vessel diameter.
2. Intervention characteristics: types of catheters, route of access (eg, subcutaneous tunnelled catheter), access site, experienced versus inexperienced (including residents and fellows) operators, original Seldinger technique or any possible variations (eg, anterior wall puncture, vein transfixation, ‘catheter over a needle’ puncture, hollow needle puncture), combination of methods.

**Table 3:**
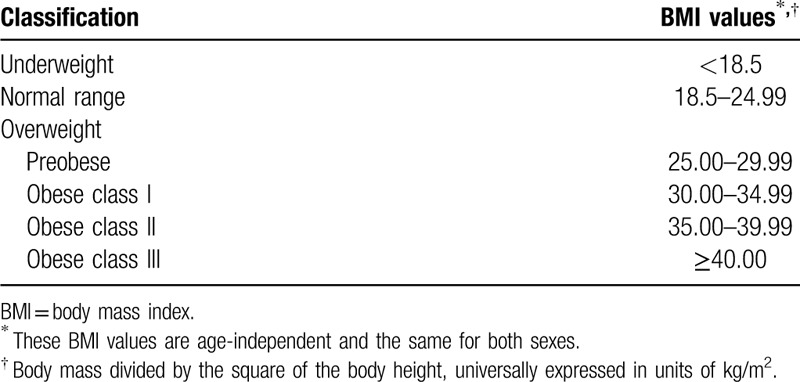
Classification of adults according to BMI.

Since we are planning to explore possible causes of substantial heterogeneity with subgroup analysis (assessment of heterogeneity), we will use all outcomes in subgroup analyses.

We will use the formal test for subgroup differences in RevMan 5.3,^[18]^ and base our interpretation on this.

### 3.14 Sensitivity analysis

We plan to carry out the following sensitivity analyses, to test whether key methodological factors or decisions have affected the main result:

1. We will consider the overall risk of bias of an included study as low if there are no high-risk judgments in the 4 domains of random sequence, allocation concealment, incomplete outcome data, and selective reporting. After that, we will perform analysis including only studies with a low risk of bias.
2. We will examine both the fixed-effect model and random-effects model meta-analyses, and we will explore any differences between the 2 estimates. These results will be presented and compared with the overall findings.

## 4 Conclusions

We will base our conclusions only on findings from the quantitative or narrative synthesis of included studies for this review. We will avoid making recommendations for practice and our implications for research will suggest priorities for future research and outline what the remaining uncertainties are in the area.

## 5 Discussion

Although there are a great number of techniques for central intravenous access, there is a lack of high-quality evidence to decide which one is better in terms of cost-effectiveness. PICC is a recent technique with uncertain results comparing its effectiveness with CVC. Johansson et al^[14]^ proposed that PICC is more related to deep venous thrombosis and less related to catheter occlusion than traditional CVC. However, this statement is not consensual in the literature. Therefore, this SR is particularly important and will attempt to address this lack of robust evidence.

## Author contributions

**AKPS**: drafting the protocol; will analyse and interpret data; draft the review and future review updates

**FCFA**: drafting the protocol; will analyse and interpret data; draft the review and future review updates

**FKYS**: drafting the protocol; will select trials; extract, analyse and interpret data; draft the review and future review updates

**HJGN**: drafting the protocol; will analyse and interpret data; draft the review and future review updates

**JEA**: drafting the protocol; will analyse and interpret data; draft the review and future review updates

**LCUN**: drafting the protocol; will arbitrate any disagreement in trials’ selection and risk of bias judgements, will analyse and interpret data; draft the review and future review updates

**LL**: drafting the protocol; will analyse and interpret data; draft the review and future review updates

**RBA**: drafting the protocol; will analyse and interpret data; draft the review and future review updates

**RLGF**: drafting the protocol; will select trials; extract, analyse and interpret data; draft the review and future review updates

**VTV**: drafting the protocol

## References

[1] Ullman AJ Marsh N Mihala G Complications of central venous access devices: a systematic review. Pediatrics 2015;136:e1331–44. 26459655 10.1542/peds.2015-1507

[2] Moureau N Poole S Murdock MA Central venous catheters in home infusion care: outcomes analysis in 50,470 patients. J Vasc Interv Radiol 2002;13:1009–16. 12397122 10.1016/s1051-0443(07)61865-x

[3] Miller DL O’Grady NP Guidelines for the prevention of intravascular catheter-related infections: recommendations relevant to interventional radiology. J Vasc Interv Radiol 2003;14 (2 pt 1):133–6. 12582182 10.1016/s1051-0443(07)60120-1

[4] Fearonce G Faraklas I Saffle JR Peripherally inserted central venous catheters and central venous catheters in burn patients: a comparative review. J Burn Care Res 2010;31:31–5. 20061834 10.1097/BCR.0b013e3181cb8eaa

[5] Petree C Wright DL Sanders V Reducing blood stream infections during catheter insertion. Radiol Technol 2012;83:532–40. 22763830

[6] Armstrong SD Thomas W Neaman KC The impact of antibiotic impregnated PICC lines on the incidence of bacteremia in a regional burn center. Burns 2013;39:632–5. 23010088 10.1016/j.burns.2012.08.017

[7] Evans M Ryder MA Invited Review: vascular access devices: perspectives on designs, complications, and management. Nutrition in Clinical Practice 1993;8:145–52. 8289767 10.1177/0115426593008004145

[8] Ng PK Ault MJ Ellrodt AG Peripherally inserted central catheters in general medicine. Mayo Clin Proc 1997;72:225–33. 9070197 10.4065/72.3.225

[9] Moran J Colbert CY Song J Screening for novel risk factors related to peripherally inserted central catheter-associated complications. J Hosp Med 2014;9:481–9. 24911379 10.1002/jhm.2207

[10] Turcotte S Dubé S Beauchamp G Peripherally inserted central venous catheters are not superior to central venous catheters in the acute care of surgical patients on the ward. World J Surg 2006;30:1605–19. 16865322 10.1007/s00268-005-0174-y

[11] Chopra V Anand S Krein SL Bloodstream infection, venous thrombosis, and peripherally inserted central catheters: reappraising the evidence. Am J Med 2012;125:733–41. 22840660 10.1016/j.amjmed.2012.04.010

[12] Schmidli J Widmer MK Basile C Editor's choice—vascular access: 2018 Clinical Practice Guidelines of the European Society for Vascular Surgery (ESVS). Eur J Vasc Endovasc Surg 2018;55:757–818. 29730128 10.1016/j.ejvs.2018.02.001

[13] Guyatt GH Oxman AD Vist GE GRADE: an emerging consensus on rating quality of evidence and strength of recommendations. BMJ 2008;336:924–6. 18436948 10.1136/bmj.39489.470347.AD PMC2335261

[14] Johansson E Hammarskjöld F Lundberg D Advantages and disadvantages of peripherally inserted central venous catheters (PICC) compared to other central venous lines: a systematic review of the literature. Acta Oncol 2013;52:886–92. 23472835 10.3109/0284186X.2013.773072

[15] Higgins JPT Thomas J Chandler J Cochrane Handbook for Systematic Reviews of Interventions version 6.0 (updated July 2019). Available at: www.training.cochrane.org/handbook. Published 2019. Accessed December 11, 2019.

[16] Shamseer L Moher D Clarke M Preferred reporting items for systematic review and meta-analysis protocols (PRISMA-P) 2015: elaboration and explanation. BMJ 2015;350:g7647. 25555855 10.1136/bmj.g7647

[17] Ware JE Sherbourne CD The MOS 36-item short-form health survey (SF-36). I. Conceptual framework and item selection. Med Care 1992;30:473–83. 1593914

[18] Review Manager. Review Manager (RevMan) [Computer Program]. Version 5.3. Copenhagen: The Nordic Cochrane Centre, The Cochrane Collaboration, 2014. Copenhagen: The Cochrane Collaboration; 2014.

[19] Higgins JPT Savović J Page MJ Elbers RG Sterne JA Chapter 8: Assessing risk of bias in a randomized trial. /handbook/current/chapter-08. Accessed December 11, 2019.

[20] Schünemann H Vist GE Higgins JPT Chapter 15: Interpreting results and drawing conclusions. /handbook/current/chapter-15. Accessed December 11, 2019.

[21] Deeks J Higgins JPT Altman DG Chapter 10: Analysing data and undertaking meta-analyses. In Higgins JPT, Thomas J, Chandler J, Cumpston M, Li T, Page MJ, Welch VA (editors). Cochrane Handbook for Systematic Reviews of Interventions version 6.0 (updated July 2019). /handbook/current/chapter-10. Accessed December 11, 2019.

[22] GRADEpro. GRADEpro. Available at: https://gradepro.org/cite/. Accessed December 11, 2019.

[23] Reinhardt U Cheng T The world health report 2000—Health systems: improving performance. Bull World Health Organ 2000;78:1064.

